# The value of the patient and public contribution to cancer research UK’s review of covid-19 impact on its clinical research portfolio

**DOI:** 10.1186/s40900-021-00279-w

**Published:** 2021-06-01

**Authors:** Anne Croudass, Richard Stephens

**Affiliations:** 1 CRUK Lead Research Nurse, 2 Redman Place, London, E20 1JQ UK; 2 Patient and Public Representative, 18 Russell Close, Stevenage, PB SG2 8 UK

**Keywords:** Patient/public contributors, Cancer, Clinical trials, Funding, Review

## Abstract

**Background:**

In July 2020 Cancer Research UK undertook a rapid review of the studies in its clinical research portfolio to assess the impact of the Covid-19 pandemic. The review examined over 160 research studies funded by the charity, and in keeping with its usual practice, the charity involved patient/public contributors in the review process.

**Main body:**

Cancer Research UK (CRUK) spends over £450 million pa on research, including clinical trials, tissue collections, laboratory science and biomarker studies. It has involved patient/public contributors in clinical research funding decisions for ten years, recruiting volunteers from the National Cancer Research Institute’s (NCRI) Consumer Forum. The NCRI is a partnership of funders, including the 4 UK governments and major charities such as CRUK. Its Consumer Forum is a group of volunteers with personal experience of cancer as patients or carers, who are trained for and experienced in working on national strategic bodies as well as on individual research studies.

The CRUK whole-portfolio review was held over a two-week period in a series of online meetings. A pair from the team of patient/public contributors was included in each meeting, and they made comments on every application reviewed as well as participating in reaching decisions.

**Conclusions:**

The process not only demonstrated CRUK’s continued commitment to involving patient/public contributors in their funding decisions, but also provided an opportunity for these contributors to take a holistic view of processes to inform future patient/public contribution in the charity’s work, as well as to influence the decisions about the individual studies being reviewed.

## Plain English summary

In July 2020 Cancer Research UK undertook a rapid review of the studies in its clinical research portfolio to assess the impact of the Covid-19 pandemic. The review examined over 160 research studies funded by the charity, and in keeping with its usual practice, the charity involved patient/public contributors in the review process.

Cancer Research UK (CRUK) spends over £450 million pa on research, including clinical trials, tissue collections, laboratory science and biomarker studies. It has involved patient/public contributors in clinical research funding decisions for ten years, recruiting volunteers from the National Cancer Research Institute’s (NCRI) Consumer Forum. The NCRI is a partnership of funders, including the 4 UK governments and major charities such as CRUK. Its Consumer Forum is a group of volunteers with personal experience of cancer as patients or carers, who are trained for and experienced in working on national strategic bodies as well as on individual research studies.

The CRUK whole-portfolio review was held over a two-week period in a series of online meetings. A pair from the team of patient/public contributors was included in each meeting, and they made comments on every application reviewed as well as participating in reaching decisions.

The process not only demonstrated CRUK’s continued commitment to involving patients and the public in their funding decisions, but also provided an opportunity for these contributors to take a holistic view of processes to inform future patient/public contributionin the charity’s work, as well as to influence the decisions about the individual studies being reviewed.

## Background

Clinical trials play an essential role in determining the effectiveness and safety of new cancer drugs, and in the process provide patients with access to potentially life-saving new treatments still early in development. Cancer Research UK (CRUK) funds nearly 200 clinical studies at any one time in order to make progress in achieving the charity’s ambition that by 2034, 3 in 4 patients will survive their cancer by ten or more years [1].

In July 2020 Cancer Research UK undertook a rapid review of the studies in its clinical research portfolio. This review was in response to the challenges posed by the initial impact of the Covid-19 pandemic, which included an almost blanket suspension of recruitment to non-Covid clinical trials by sponsors, investigators and study sites, redeployment of National Health Service (NHS) and laboratory staff, the closure of university laboratories, some changes to standard of care and for many patients, reduced access to resources and services. The purpose of the review was to assess the impact of Covid-19 on these clinical trials and on the infrastructure supporting them and to establish whether they would still be viable in a “post-Covid” world.

Whilst it was not a criteria for judgements in the review, the Covid-19 pandemic had already significantly affected charitable income. Therefore it was important to understand which studies would be able to complete recruitment, how much delay there might be, and if the finished study would then still contribute to CRUKs strategic aims within the context of a more limited budget to support research in the short-term future.

**Fig. 1:**
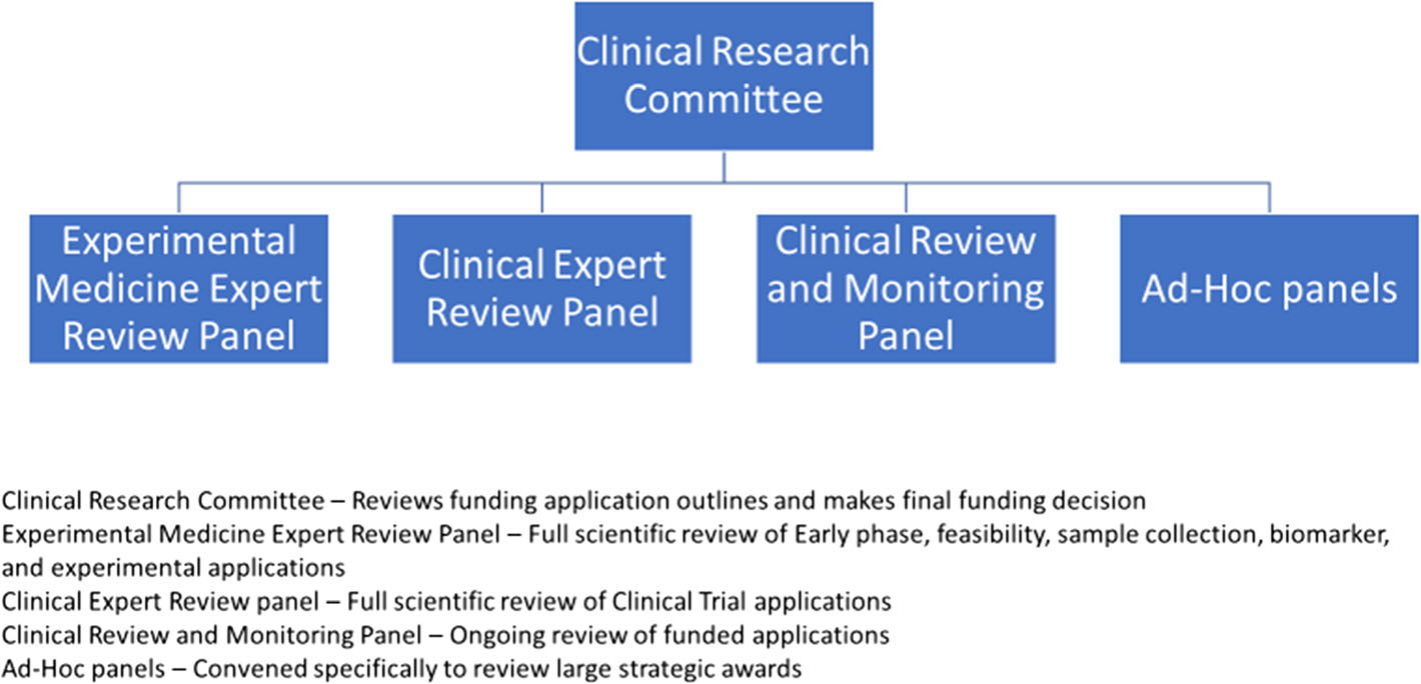
CRC and associated panels

Since 2011 CRUK has routinely involved patient/public contributors in the funding decisions made by its Clinical Research Committee (CRC) and various feeder panels (Fig. 1), and the rapid portfolio review was no exception. With the potential impact of Covid 19 on cancer patients, care and clinical research, having the patient’s perspective was essential to the portfolio review process, as decisions made as part of the review could potentially have an impact on those patients already recruited to trials, and might also affect future cancer patients.

## Main body

### Cancer Research UK

CRUK is the world’s largest charity dedicated to saving lives through research. In 2019/20 CRUK spent £455 million on research, including laboratory based science, prevention, clinical trials and infrastructure awards. CRUK works with over 150 hospital trusts, supports over 4000 Researchers, Doctors and Nurses and funds up to 200cancer research studiesacross all cancer sites [2].

### The review process

The review included 163 current clinical studies of the 180 funded or endorsed through the CRC (Table 1). These included clinical trials sample collections, experimental medicine awards and biomarker awards. The 17 studies that were not included had either completed all follow up and were in the write-up stage or were biomarker projects that were funded by CRUK and did not rely on NHS resources. At the time of the review, these were deemed unlikely to be directly affected by the impact of Covid-19.

**Table 1:** Trials included in the review

Award Type	In set up	Open to recruitment	Closed to recruitment/in follow up	Total
**Clinical trial award**	28	60	49	137
**Sample Collection**	1	8	7	17
**Biomarker project**	0	5	0	5
**Experimental Medicine**	2	2	0	4
**Total**	31	75	56	163

Review meetings were held using the Microsoft Teams platform, and focused on the progress or otherwise of the studies up to the end of May 2020 (ie 4–5 weeks before the review), their likely new reporting dates and the continued relevance of the findings at that point. The review was held over several days, with studies being grouped to reflect the topic areas of the National Cancer Research Institute (NCRI) Research Groups (RG) (Table 2).

**Table 2:** The National Cancer Research Institute and Research Groups

The NCRI was established in 2001 as a partnership to drive collaboration between organisations that fund cancer research. Cancer Research UK is one of the NCRI Partners. The NCRI aims to deliver better outcomes for all those affected by cancer throughout the UK and beyond. Bringing together the brightest scientific and medical minds to advance cancer research in the UK, they identify gaps in cancer research and address them, as well as looking out for unnecessary, expensive duplication of effort and guard against it [3]. The NCRI Research Groups bring together clinicians, scientists and patients, carers and family members, amongst many others to coordinate the development of a strategic portfolio of trials. They have been in existence for more than fifteen years and are a central part of the UK’s cancer research infrastructure. This includes considering new research questions, developing proposals for trials, securing funding and overseeing the portfolio of existing studies. These Research Groups are multi-disciplinary, and typically have 15–20 Scientific members and (at least) 2 consumer members to assist the group in understanding the perspectives of patients, carers and the public that are relevant to the work of the Research Group. The Research Groups liaise with funding bodies in order to secure funding for their trial portfolio as well as providing proposals guidance to improve the quality of cancer research, and especially clinical trials. They interact with the Clinical Trials Units and UK clinical research networks (including the devolved nations) to optimise delivery of trials as well as other site-specific and cross-cutting NCRI Groups as appropriate. NCRI Research Groups meet twice a year with meetings of subgroups or workstreams held in between. These groups also have NCRI Consumers sitting on them. The main Research Groups are supported by NCRI which provides strategical, managerial and organisational support [4].	

Each review session was undertaken by a panel of between 12 and 15 members, including scientists, clinicians, statisticians and patient/public contributors, as is usual for CRUK clinical research funding committees. In addition, for this whole-portfolio review the NCRI RG Chairs or their nominated representative attended relevant sessions, so that the recommendations of the panel took account of the particular NCRI RG’s strategic priorities for their national portfolios.

### The role of patient and public contributors

CRUK has had patient/public representation on its funding committees for over 10 years. Initially there was one contributor, on the main funding committee only, and the role was as an observer to ensure due process. The role and remit have evolved so that there are now two patient/public contributors on each panel and committee. They are full voting members, and as such they have equal scoring rights, are bound by the same confidentiality, governance and conflict requirements and are offered the same honoraria as other members. They comment on each application under consideration. They provide the perspective of people affected by cancer on applications, considering areas including but not limited to, the patient acceptability of study design, the value of the study aims to the intended patient populations and whether there are any potential unexpected adverse effects from the point of view of the participants. They also provide insight and advice on the level of patient and public involvement in the applications received.

All the current CRUK patient/public committee members are, or have previously been, active members of the NCRI Consumer Forum (Table 3). This membership is extremely beneficial as it confers the level of understanding, training and professionalism required to contribute fully at CRUK (and other) strategic research meetings. The patient/public contributors involved in the rapid portfolio review were those who also sit on the Panels and Committees shown in Fig. 1.

**Table 3:** The NCRI Consumer Forum

The NCRI Consumer Forum fosters a vibrant and collaborative community to work with the NCRI as partners in cancer research. The Forum offers space for mutual learning and exchanging knowledge and expertise in a coordinated way as well as providing a space for peer support. Members of the NCRI Consumer Forum also sit on NCRI Research Groups, subgroups and workstreams, and as such are required to contribute to group activities by attending and actively participating in meetings [5]. NCRI Consumers who sit on Research Groups are recruited in open national competition and receive training for their roles. A crucial part of that role is to assist the NCRI Research Group in understanding the perspectives of patients, carers and the public where relevant to the work of the group [6].	

This whole-portfolio review was a new experience for the team at CRUK and for the patient/public contributors. Working with their mentor (CRUK’s lead research nurse), the contributors were assigned in pairs to cover up to 3 of the 6 review meetings each. For the Paediatric/Teenager and Young Adult review, patient/public contributors with specific experience in this field were recruited from the NCRI Consumer Forum. For the other 5 review meetings, all the contributors had had previous experience of working with CRUK funding committees or other CRUK research initiatives. The contributors knew each other and had worked together previously, which for this task was another benefit.

Debbie Keatley, PPI representative stated,

“To be honest, the request from CRUK for public members to be involved in the reviews that took place in summer 2020 felt daunting. This felt very different to funding and monitoring meetings in the course of an ordinary year and I was honoured to take part but under the circumstances it could not be anything but a difficult process.”

As this review was different to the standard funding committee meetings, a new template form was developed by CRUK and the patient/public contributors to guide and capture their feedback (Table 5 in Appendix). This reflected the questions asked of those submitting the trial paperwork for review and provided consistency across each meeting. For each study discussed, 3 lead reviewers were nominated; a clinician, a statistician and a patient/public contributor, ensuring that the patient view was given equal consideration to the scientific views. After each meeting there was opportunity for a debrief with theirmentor, where the contributors could reflect on the meeting and make suggestions that would improve their experience of the process for subsequent meetings.

## Discussion

Involving patient/public contributors in this review demonstrates CRUKs commitment to putting patients at the heart of all that the charity undertakes. It was evident to CRUK staff and participating researchers that the patient/public contributors were adding a unique and essential expertise. Without exception they were well prepared and engaged. They were flexible and accommodating to the tight schedules, new technological requirements and evolving time frames, giving concise, thoughtful and objective feedback throughout. Most importantly the patient voice was not only heard but carried equivalent weight to that of other panel members.

Mat Baker commented,

“I was delighted to have the opportunity to contribute to this research review and to the future of so many potentially practice changing trials. It was important to ensure that the patient perspective was clearly articulated, and I was pleased that its value in the decision-making process was so positively acknowledged.”

The value of a pool of patient/public contributors with appropriate skills to respond at short notice and to contribute effectively to the review validated the CRUK stance that for this type of strategic meeting and decision-making process, patient/public contributors representatives should ideally have a background understanding of the research environment, such as that provided by NCRI involvement.

Ian Walker, Director of Research Funding, Communications and Partnerships said,

“As always, the comments from our patient contributors were insightful, thoughtful and added great value to the discussions. The insights and intelligence we have gathered through the process will provide us with really important data to support both our research agenda and our policy priorities going forward.”

The portfolio review had to make difficult decisions about the future of clinical trials. Involving patient/public contributors in these decisions gives credibility to those decision and outcomes, for the cancer community as a whole and in particular for people affected by cancer, especially those participating in research studies.

Paula Ghaneh, Professor of Surgery, University of Liverpool and Chair of the Upper GI and Colorectal review meeting commented,

“The patient contribution to the portfolio review was and continues to be extremely valuable. In all manner of committee meetings, they always manage to sum up the key issues in a clear and precise way. With the ethos of CRUK at the centre of their arguments, they remind us of all the people who raise the money for CRUK and what really matters for patients. They always give us the perspective to make the best decisions even if they are difficult or tough.”

A further benefit of involving patient/public contributors in the review was the identification of cross cutting themes. Four of the contributors attended at least three review sessions each, whereas the majority of the other panellists attended only one or two. Moreover, the patient/public contributors worked together informally during the review and more formally afterwards to identify themes for CRUK and for other patient/public contributors to consider for the future. These included

- the need for robust remote assessment processes
- the involvement of primary care in delivering protocol-led care
- the need to update patient information to reflect impact of Covid-19 and the opportunity to incorporate electronic consenting procedures.

The patient/public contributors also collated a report for their NCRI Consumer colleagues. As well as providing an overview of the process and outcomes, it included reflections on how their involvement in this review could benefit and inform wider NCRI consumer activity (Table 4). By circulating the report to all 100 members of the NCRI Consumer Forum, they encouraged other patient/public contributors to discuss, debate and disseminate the information in the report.

**Table 4:** Thoughts and ideas from patient/public contributors

Research Group Portfolios:	
1. This review was about the big picture in July; but what does it look like for September and onwards? Is there an agenda item for your next RG/subgroup/workstream meeting?	
2. In particular are there any studies where returning to previous recruitment levels may be problematic, and if so, are the issues at particular sites or is there a more general challenge for patients which you may be able to address (eg by promoting the restarted study across personal networks)?	
Current Studies:	
3. Is your study being reviewed in November or next April, and are there actions to be taken in preparation? Many of the studies reviewed in July had very optimistic new timelines; are they realistic as we move into autumn?	
4. Is it worth advising the funder now that a costed or no-cost extension may be needed? If the study is finished, what is the timescale for analysis and write-up? Does that include the QoL measures or just the primary/intervention outcomes?	
5. For studies where standard-of-care may have changed, how temporary is it? If the change is likely to be permanent, will the study results still be relevant? Will the study still recruit if the “standard” arm is no longer the same as when the study was designed? Does the randomisation need changing in ratio? Will some sites drop out, with the implications for patient choice?	
Doing Things Differently:	
6. Do the ad hoc Covid-19 arrangements offer new opportunities and/or challenges for redesigning current studies and designing new ones, in particular offering patient choice on remote consent, remote follow-up, and moving QoL online?	
7. Should academic studies look more closely at industry’s adoption of wearables and apps to record real-time PROMs and real-world data?	
8. Would any of these changes help address current issues of disadvantage and under-representation? Or might they disadvantage different groups?	
9. Are there now opportunities for sub-studies to address the NCRI’s patient-derived Living With and Beyond Cancer research priorities? https://www.ncri.org.uk/lwbc/ Should QOL/PROMs that include psychosocial elements/experiences become standard in all trials, and if so, which measures?	
10. What opportunities does the restart offer to embed research choices into patient/clinician conversations, more than they already were, as recommended in the NCRI Consumer Forum’s 2012 report “Action on access” https://www.ncri.org.uk/wp-content/uploads/2012-NCRI-Action-on-access-report.pdf	

This was a further demonstration of the value of having patient/public contributors linked into NCRI consumer activities and thence to their own national, international, local and online networks of patient representatives and groups. Mat Baker observed,

“CRUK have once again demonstrated that they are at the forefront of good practice in involving patients and carers at the heart of the research decision making process. A necessary corollary is that patients and carers possess the knowledge and skills to contribute effectively at this level. Fortunately the NCRI Consumer Forum, through its collaborative ethos and exacting standards, enables patients and carers such as myself to step up and to forge partnerships with the leading teams in cancer research.”

## Conclusion

The novel nature of the review for both CRUK staff and committee members provided equal opportunity for patient/public contribution to the discussions and in the decisions. This increased the levels of engagement and responsibility of the patient/public contributors,, demonstrated their ability to provide useful and relevant input and kept patients at the centre of the process. As Debbie Keatley said,

“This was an extraordinary series of reviews, brought about by extraordinary events but at the end of it all were real patients, and for some, taking part in *their* trial offered access to otherwise unavailable treatments and not being able to take part carried real consequences. It was sobering to absorb how hard research teams had worked to keep trials open wherever possible, to adjust protocols, to attempt to keep as much valuable work and learning as possible and to restart as soon as possible. It was clear that CRUK staff too had worked extremely hard to support trials, and us - a resource intensive process, providing us with rich information and context. The impact of COVID-19 on clinical research will be felt for a long time but we found many examples of good practice under very difficult conditions and the recommendations we made were taken together, equally, with outcomes for current and future patients held firmly in mind.”

This review has provided CRUK with a further opportunity to develop the patient/public contributor role in funding committees and has prompted a review of the patient/public contribution to funding committee practices. This will lead to a piece of work to further strengthen the patient/public contribution, including increasing the number of patient/public contributors involved, and will be developed jointly by staff and patient/public contributors.

CRUK would like to thank all the patient/public contributors, Mat Baker, Debbie Keatley, Angela Polanco, Janette Rawlinson, Richard Stephens and Max Williamson for their valuable input to the review.

## Data Availability

Not Applicable.

## Appendix

**Table 5:** CRC Portfolio Review - Patient/public review form

Disease site	
Date	
PPI rep	

This review will encompass trials in set up, trials that were open to recruitment pre-COVID19 and trials in follow up

Please complete this form for proposals if you have comments that you would like to be considered as part of the applicant feedback. You do not have to complete this form for all studies if not relevant or you have no comments

The questions below are to give you guidance, but you can comment on any aspect of the study you feel relevant

For trials in set up

**Table:**

Study name	
Can the study continue as originally planned? Please consider the impact of the pandemic on patients’ safety, Inclusion/Exclusions criteria, deliverability in post-COVID19 NHS.	
Do you think the potential impact of the study for cancer care and cancer patients has changed? Will this be practice changing?	
Do you think the study will still be of interest to patients, and is it likely to recruit?	
Will the study need revised/additional PPI to start?	
What would be the ethical considerations/ implications for patients if the study did not go ahead?	
Is this study still value for money in light of budget reduction?	
Any other comments	

For trials open to recruitment

**Table:**

Study name	
Could this trial restart as per protocol? Please consider the impact of the pandemic on patients’ safety, Inclusion/Exclusion criteria, changes to standard of care arms if applicable, and deliverability in post-COVID19 NHS	
Impact of Covid on recruitment.	
Do you think the study will still be of interest to patients, and is it likely to continue recruiting?	
Has the potential impact of the study for cancer care and cancer patients been affected? Is this practice changing?	
Will the study need revised/additional PPI to restart?	
What would be the ethical considerations/ implications for patients if the study was discontinued? Would anything be lost if this study closed and reported now on the data available?	
Is the study still value for money in light of budget reduction	
Any other comments	

For trials in follow up

**Table:**

Will the follow up schedule for this study need to change? Please consider patients’ safety, alternative methods of obtaining follow-up data, financial implications of changing the follow-up schedule.	
Could the study follow-up be stopped ow?	
Is any additional PPI input required?	
What would be the cost/benefit of completing follow up?	
What would be the ethical considerations/ implications for patients if the study stopped early?	
Any other comments	

## Abbreviations

CRC: Clinical Research Committee
CRUK: Cancer Research UK
NCRI: National Cancer Research Institute
NHS: National Health Service
RG: Research Group
